# Does bevacizumab impact anti-EGFR therapy efficacy in metastatic colorectal cancer?

**DOI:** 10.18632/oncotarget.7008

**Published:** 2016-01-25

**Authors:** Valentin Derangère, Jean David Fumet, Romain Boidot, Leila Bengrine, Emeric Limagne, Angélique Chevriaux, Julie Vincent, Sylvain Ladoire, Lionel Apetoh, Cédric Rébé, François Ghiringhelli

**Affiliations:** ^1^ INSERM, U866, Faculté de Médecine, Université de Bourgogne, Dijon, France; ^2^ Centre Georges-François Leclerc, Dijon, France

**Keywords:** metastatic colon cancer, anti-EGFR therapy, bevacizumab, Stat-3, VEGFR

## Abstract

Anti-EGFR therapy and antiangiogenic therapies are used alone or in combination with chemotherapies to improve survival in metastatic colorectal cancer. However, it is unknown whether pretreatment with antiangiogenic therapy could impact on the efficacy of anti-EGFR therapy.

We selected one hundred and twenty eight patients diagnosed with advanced colorectal cancer with a *KRAS* and *NRAS* unmutated tumor. These patients were treated with cetuximab or panitumumab alone or with chemotherapy as second or third-line. Univariate and multivariate Cox model analysis were performed to estimate the effect of a previous bevacizumab regimen on progression free survival and on overall survival during anti-EGFR therapy. *In vitro* studies using wild type *KRAS* and *NRAS* colon cancer cells were performed to evaluate the impact of VEGF-A on cetuximab-induced cell death.

The median progression free survival (PFS) during anti-EGFR treatment was significantly different between the bevacizumab group and the non-bevacizumab group (2.8 and 4 months respectively; *p* = 0.003). The median overall survival from the beginning of the metastatic disease was similar in the two groups (41.3 and 42 months respectively; *p* = 0.7). *In vitro*, VEGF-A induced a resistance toward cetuximab cytotoxicity on three *KRAS* and *NRAS* wild type colon cancer cell lines in a VEGFR2 and Stat-3-dependent manner.

All in all, our clinical data, supported by *in vitro* procedures, suggest that a previous anti-VEGF therapy decreases anti-EGFR efficacy. Although these results are observed in a limited cohort, they could be taken into consideration for a better strategy of care for patient suffering from metastatic colorectal cancer.

## INTRODUCTION

Colorectal cancer is the second cause of cancer death worldwide [1]. Approximately 30% of patients with colorectal cancer have an overt metastatic disease at diagnosis. When all metastatic sites could not be surgically removed, treatment remains palliative and requires different chemotherapeutic protocols. For patients with non-operable metastatic colorectal cancer (mCRC), there is no curative option. However, the use of palliative systemic chemotherapy dramatically enhances response rates, progression-free survival (PFS) and overall survival (OS) [2–4]. In a recent phase III clinical trial of palliative chemotherapy, the overall survival of patients has reached 24 to 30 months [5, 6]. Colorectal cancer treatment is currently based on the use of three cytotoxic chemotherapy, fluoropyrimidine, oxaliplatin and irinotecan associated with targeted therapies (anti-Epithelial Growth Factor Receptor (EGFR) (panitumumab and cetuximab) or anti-Vascular Endothelium Growth Factor (VEGF) (bevacizumab or aflibercept) monoclonal antibodies). However, the treatment of incurable mCRC remains currently a challenging question. The use of antiangiogenic agents as first and second-line was shown to improve overall survival [7, 8]. Recently, clinical trials underlined that permanent antiangiogenic blocking as first and second-line improved overall survival [9]. The use of anti-EGFR therapy could also improve survival as first or third-line treatment [10–12]. In addition, the use of anti-EGFR therapy was rationalized using genomic testing of Kirsten Rat Sarcoma *(KRAS)* and Neuroblastoma RAS *(NRAS)* mutation status. Indeed, these assays provide a better selection of patients carrying wild-type tumor assuring optimal response to anti-EGFR therapy and avoiding an inappropriate use of this targeted therapy when KRAS and/or RAS were mutated [13]. In addition, recent advances in management of classical cytotoxic agents underline the possibility to administrate the three cytotoxic drugs as first-line of colorectal cancer treatment [14, 15].

While all these treatments improve overall survival, the optimal sequence of therapy still needs to be determined. Anti-EGFR therapy was first designed for patients who developed resistance to chemotherapy, explaining why it is frequently used as second or third-line. In such case it is not known if a previous antiangiogenic administration could modify the efficacy of anti-EGFR therapy. To address this question, we used our patients’ database treated for a metastatic colorectal cancer, with a restriction to population with current approval for anti-EGFR therapy, i.e. *KRAS* and *NRAS* wild type population.

## RESULTS

### Patients' characteristics

We selected 198 patients who received cetuximab or panitumumab as second or third-line therapy for mCRC from our cohort treated at Georges Francois Leclerc Cancer Center. We completed *KRAS* and *NRAS* genotyping for all patients and retained 128 patients with wild type status for *KRAS* and *NRAS* genes. Of these patients, 76 (59%) received bevacizumab based chemotherapy during the first- line therapy for metastatic disease. Patients and tumors characteristics are shown in Table 1. We did not observe significant difference between the two groups of patients who received bevacizumab or not in first-line for the main clinical and biological characteristics, except for age which was significantly younger in the non-bevacizumab group (59 vs 66 years, *P* = 0.02). Median follow-up at the data cut-off point was 24 months in bevacizumab group and 28 months in chemotherapy group.

**Table 1:** Patient and tumor characteristics (*n* = 128)

		Previous treatment without bevacizumab *N* = 52	Previous treatment with bevacizumab *N* = 76	Overall *N* = 128	*p*-value
**Age (year)**					
	Median (min;max)	59 [28; 83]	66 [35; 85]	62 [24; 90]	**0.02**
	Mean(sd)	60 (12)	65 (11)	63 (12)	
**Sex**					
	male	28 (54%)	45 (59%)	73 (57%)	**0.16**
	female	24 (46%)	21 (41%)	45 (33%)	
**Death**					
		38 (73%)	54 (71%)	92 (72%)	**0.79**
**WHO PS**					
	0–1	21 (40%)	30 (39%)	51 (40%)	**0.48**
	> 2	22 (42%)	38 (50%)	60 (47%)	
	Unknown	9 (8%)	8 (11%)	17 (13%)	
**Evolution**					
	Synchronous	32 (61%)	51 (67%)	90 (70%)	**0.64**
	Metachronous	20 (39%)	25 (33%)	38 (30%)	
**Primary tumor resection**					
	Yes	45 (86%)	58 (76%)	103 (80%)	**0.23**
	No	7 (14%)	18 (24%)	25 (20%)	
**Complete surgery of metastases**					
	No	26 (50%)	49 (64%)	85 (68%)	**0.14**
	Yes	26 (50%)	27 (36%)	53 (32%)	
**Localization of the primary tumor**					
	Colon	30 (58%)	54 (71%)	84 (65%)	**0.41**
	Rectum	22 (42%)	22 (29%)	44 (35%)	
**Anti-EGFR line**					
	2	10 (19%)	21 (28%)	31 (24%)	**0.35**
	3	42 (81%)	55 (72%)	97 (76%)	
**number of metastatic sites**					
	1	39 (75%)	52 (68%)	91 (71%)	**0.54**
	> 1	13 (25%)	24 (32%)	37 (29%)	
**Leucocyte > 10 00/ml**					
	No	42 (81%)	61 (80%)	103 (80%)	**0.87**
	Yes	10 (19%)	15 (20%)	25 (20%)	
**Alkaline Phosphatase > 300 UI/ml**					
	No	45 (86%)	66 (87%)	111 (87%)	**0.83**
	Yes	7 (14%)	10 (13%)	17 (13%)	
**CEA level**					
	Median (min;max)	12 [0; 8235]	16 [0; 8754]	16 [0; 8754]	**0.32**
	Mean(sd)	594 (1824)	306 (1192)	428 (1485)	

### Progression free survival on anti-EGFR therapy in bevacizumab group and chemotherapy alone group in first-line treatment

**Figure 1:**
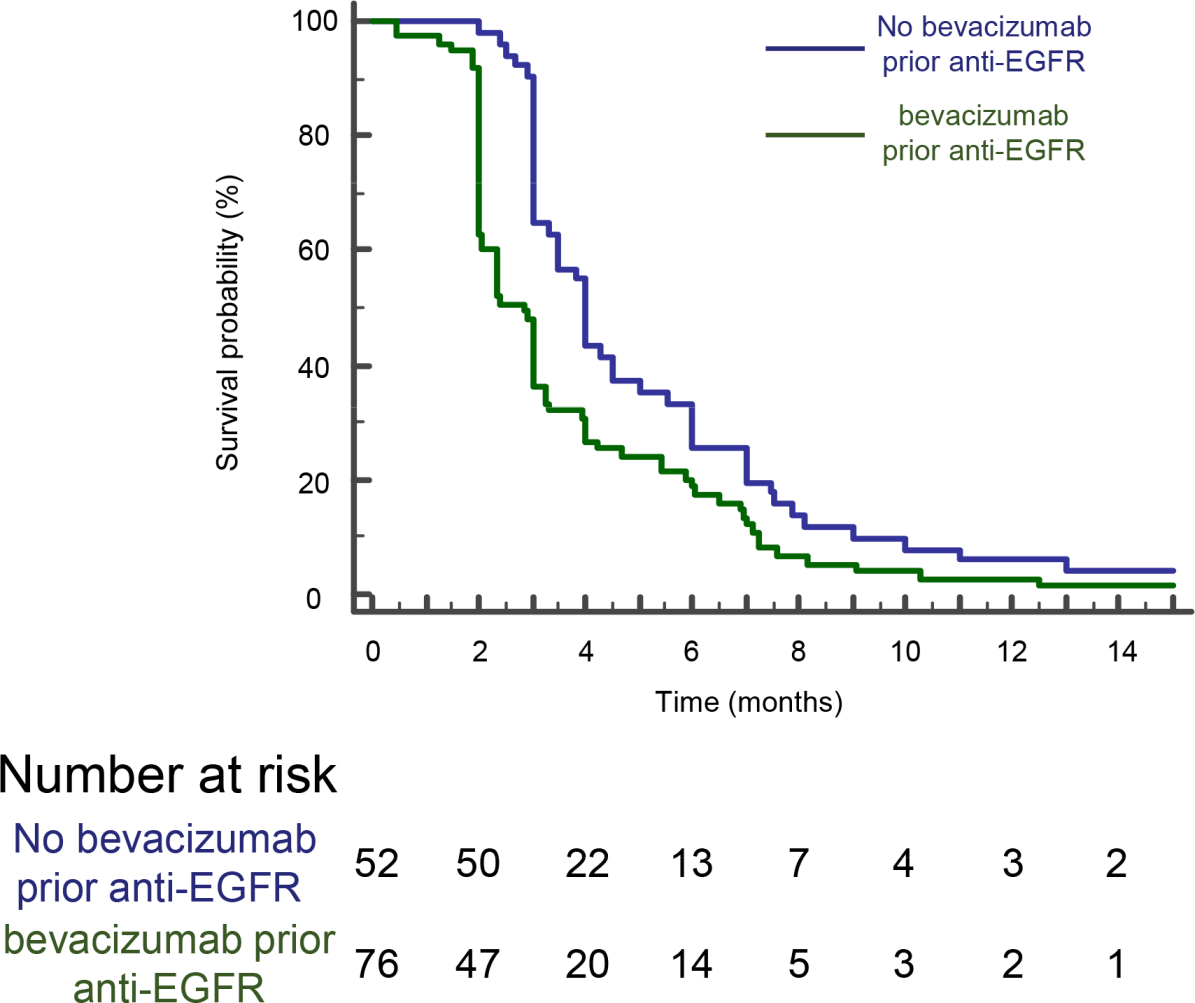
A poorer PFS is observed for patients on anti-EGFR therapy when previously treated with bevacizumab Kaplan-Meyer progression free survival curves of mCRC patients treated or not with bevacizumab prior anti-EGFR therapy. The difference was significant (*p* < 0,003, log- rank test).

All patients developed progression or death on anti-EGFR therapy. Patients receiving bevacizumab as first-line had a poorer PFS on anti-EGFR therapy compared to patients receiving chemotherapy alone (log-rank test *P* < 0.003) (Figure 1). Median PFS was 2.8 months (95% CI, 2–3 months) in bevacizumab group and 4 months (95% CI, 3.3–5 months) in non-bevacizumab group. Univariate analysis indicated that WHO performance status ≥ 2, primary tumor in place, leucocytes > 10,000/ml and previous treatment with bevacizumab are significantly associated with a poorer PFS (Table 2). Using multivariate analysis, only previous treatment with bevacizumab remained independently associated with a poorer PFS (HR = 1.7 [1.06–2.3] *P* = 0.03) (Table 2).

**Table 2:** Univariate and multivariate analysis (Cox regression) for factors associated with PFS

	Univariate analysis			Multivariate analysis		
	HR	95%CI	*p*-value	HR	95%CI	*p*-value
Age*						
	1.01					
**Sex**		[0.99; 1.03]	**0.4**			
Male	1					
Female	1.12					
CEA Level*		[0.78; 1.6]	**0.5**			
	1.001					
**WHO PS**		[0.99; 1.0002]	**0.76**			
0–1	1			1		
> = 2	1.62	[1.18; 3]	**0.04**	1.2	[0.68; 2.2]	**0.5**
**Evolution**						
Synchronous	1			1		
Metachronous	0.72	[0.5; 1.04]	**0.07**	0.82	[0.52; 1.33]	**0.4**
**Primary tumor resection**						
No	1			1		
Yes	0.56	[0.32; 0.96]	**0.005**	0.84	[0.58; 1.22]	**0.4**
**Complete surgery of metastases**						
No	1					
Yes	0.8	[0.55; 1.12]	**0.17**			
**Leucocyte > 10 00/ml**						
No	1					
Yes	1.1	[1.01; 1.75]	**0.04**			
**Alkaline Phosphatase > 300 UI/ml**						
No	1					
Yes	1.5	[0.8; 2]	**0.11**			
**Number of metastatic sites**						
1	1			1		
> 1	1.56	[1.05; 2.3]	**0.009**	1.12	[0.7; 1.7]	**0.6**
**Bevacizumab use**						
No	1	[0.8; 1.7]	**0.34**			
Yes	1.2					
**Sequence**						
No Bevacizumab before	1			1		
bevacizumab before	1.65	[1.16; 2.3]	**0.003**	1.7	[1.06; 2.8]	**0.03**

* hazard ratio for continuous variable was calculated for one unit.

### Overall survival on anti-EGFR therapy in bevacizumab group and chemotherapy alone group as first-line therapy

**Figure 2:**
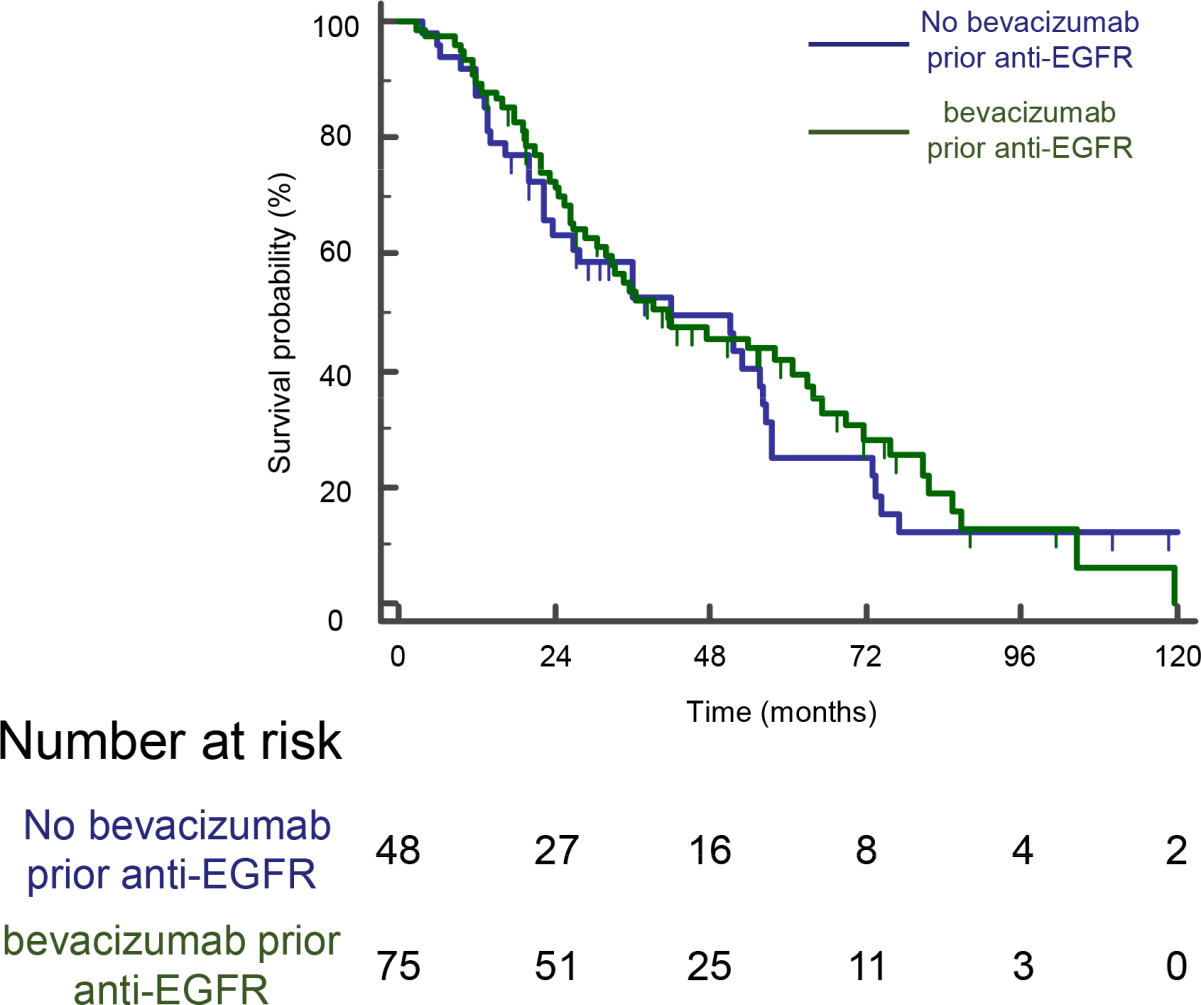
A previous bevacizumab administration have no effect on OS for patients on anti-EGFR therapy Kaplan-Meyer overall survival curves of mCRC patients treated or not with bevacizumab prior EGFR therapy. The difference was not significant (log- rank test).

Proportions of patients who died were not significantly different between the two groups, 73% in chemotherapy alone group, and 71% in bevacizumab group (*P = 0.6*). We did not detect any difference in outcome in term of OS in patients receiving bevacizumab or chemotherapy alone as first-line (log-rank test *P* < 0.7) (Figure 2). Median OS was 41.3 months (95% CI, 30.7–62.9 months) in bevacizumab group and 42 months (95% CI, 23.5–56 months) in non-bevacizumab group (*p* = 0.7). Univariate analysis indicated that high CarcinoEmbryonic Antigen (CEA) level, WHO performance status ≥ 2, synchronous metastatic disease, absence of complete metastases surgery, absence or resection of primary tumor in colon, number of metastatic site > 1, leucocytes > 10,000/ml and Alkaline Phosphatase > 300 UI/ml are significantly associated with a poorer OS. Using multivariate analysis, only performance status, previous surgery of primary tumor and phosphatase alkaline serum level remained independently associated with a poorer OS (Table 3).

**Table 3:** Univariate and multivariate analysis (Cox regression) for factors associated with OS

	Univariate analysis			Multivariate analysis		
	HR	95% CI	*p*-value	HR	95% CI	*p*-value
Age*						
	1.009	[1.0004; 1.0272]	**0.4**			
**Sex**						
Male	1					
Female	0.95	[0.62; 1.46]	**0.84**			
CEA Level*						
	1.0002	[1.0001; 1.0004]	**0.02**	1	[0.97; 1.0003]	**0.85**
**WHO PS**						
0–1	1			1		
> = 2	2.44	[2.2; 5.2]	**0.0001**	2.8	[1.2; 6.4]	**0.01**
**Evolution**						
Synchronous	1			1		
Metachronous	0.65	[0.22; 0.99]	**0.05**	1.4	[0.7; 2.8]	**0.36**
**Primary tumor resection**						
No	1			1		
Yes	0.22	[0.1; 0.53]	**< 0.0001**	0.3	[0.1;0.7]	**0.006**
**Complete surgery of metastases**						
No	1			1		
Yes	0.47	[0.3; 0.72]	**0.0004**	1.3	[0.6; 2.6]	**0.5**
**Leucocyte > 10 00/ml**						
No	1			1		
Yes	2.2	[1.15; 3.4]	**0.001**	1.3	[0.6; 2.9]	**0.5**
**Alkaline Phosphatase > 300 UI/ml**						
No	1			1		
Yes	4.1	[1.5; 11.5]	**< 0.0001**	3.9	[1.5; 10.5]	**0.005**
**Number of metastatic sites**						
1	1					
> 1	1.20	[0.6; 1.4]	**0.66**			
**Bevacizumab use**						
No	1					
Yes	0.71	[0.5; 1.2]	**0.14**			
**Sequence**						
No Bevacizumab before	1					
bevacizumab before	0.9	[0.6; 1.4]	**0.7**			

* hazard ratio for continuous variable was calculated for one unit.

### *In vitro* effect of VEGF-A on the antitumor effect of anti-EGFR

**Figure 3:**
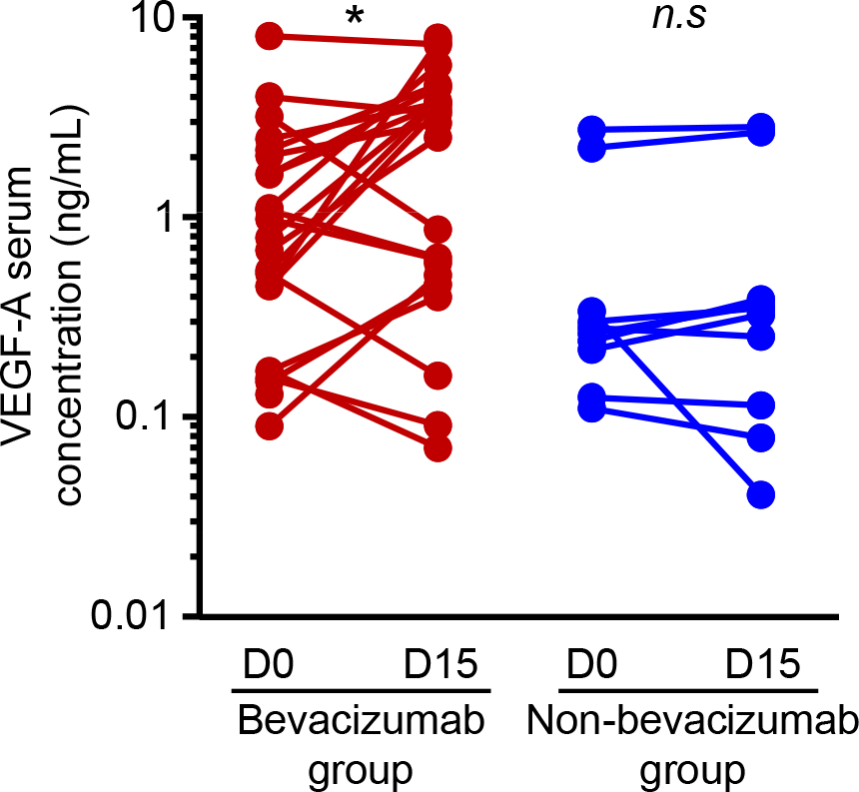
VEGF-A is increased in patients’ serum during anti-VEGF therapy VEGF-A serum level from mCRC patients (*n* = 26) treated with FOLFOX/bevacizumab chemotherapy protocol red lines) compared to patients (*n* = 12) treated with chemotherapy alone (blue lines). Assays were performed before and 15 days after bevacizumab injection and analyzed by ELISA. (*p < 0,01, *n.s*: not significant, Student *t* test).

**Figure 4:**
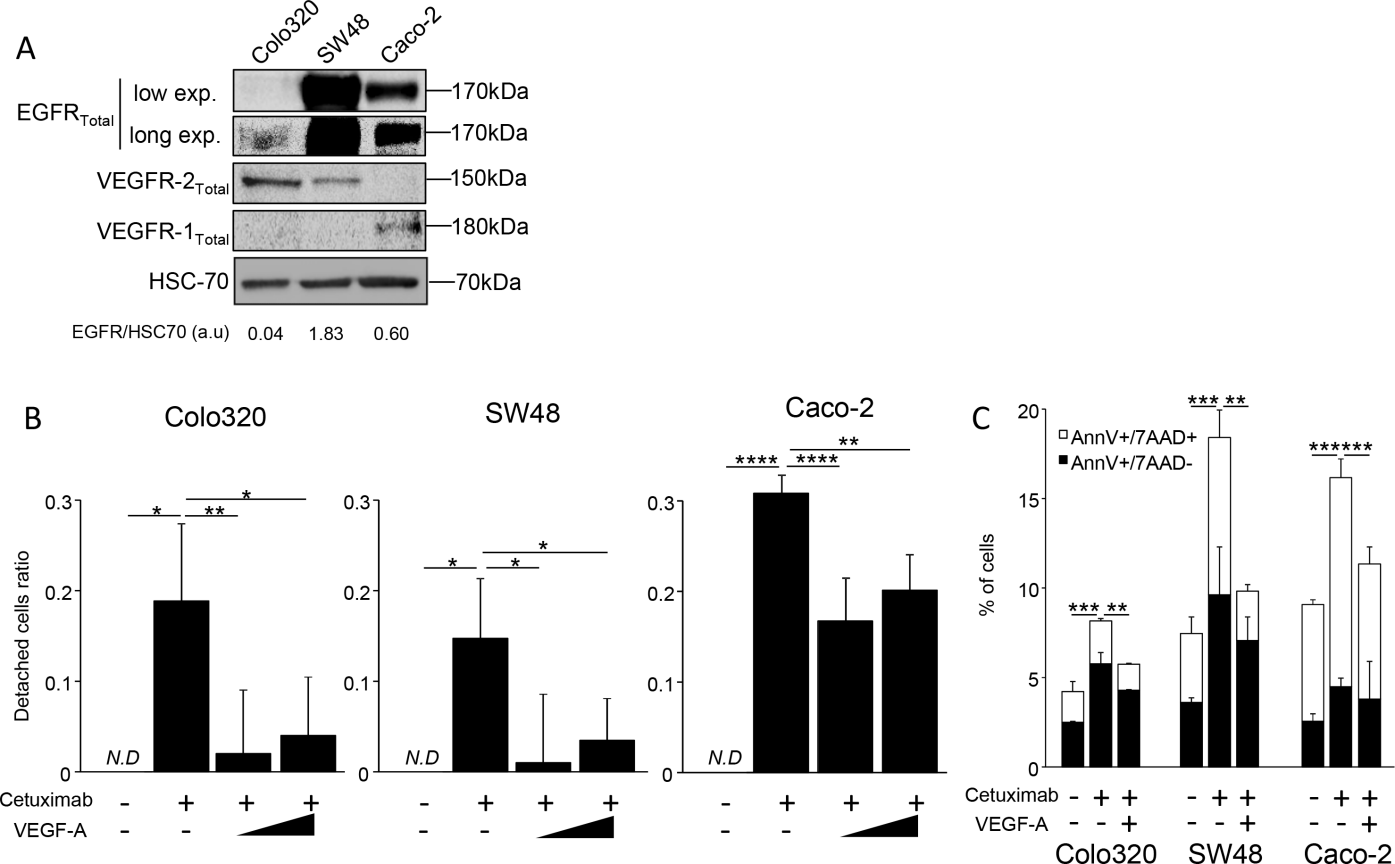
VEGF-A can inhibit cetuximab cytoxicity *in vitro* (**A**) Western Blot analysis showing VEGFR1, VEGFR2 and EGFR expression. HCS-70 was used as loading control and as a reference for EGFR quantification (a.u: arbitrary unit). (**B**) Cell proliferation analyzed by crystal violet staining. SW48, Caco-2 and Colo320 colon cancer cell lines were incubated or not with increasing dose of human recombinant VEGF-A (0,5 or 5 ng/mL). Cetuximab (500 μg/mL) was added the following day in culture medium and cell death was analyzed 7 days later. (**C**) Annexin V/7AAD staining. Cells were incubated with VEGF-A 5 ng/mL. Cetuximab 500 μg/mL was added the following day. Cell death was evaluated 24 hours after cetuximab was added, AnnexinV positive cells are in black boxes, double positive cells are in white boxes (*p < 0,1; **p < 0,01; ***p < 0,001; ****p < 0,0001; *N.D*: not determined, ANOVA test).

**Figure 5:**
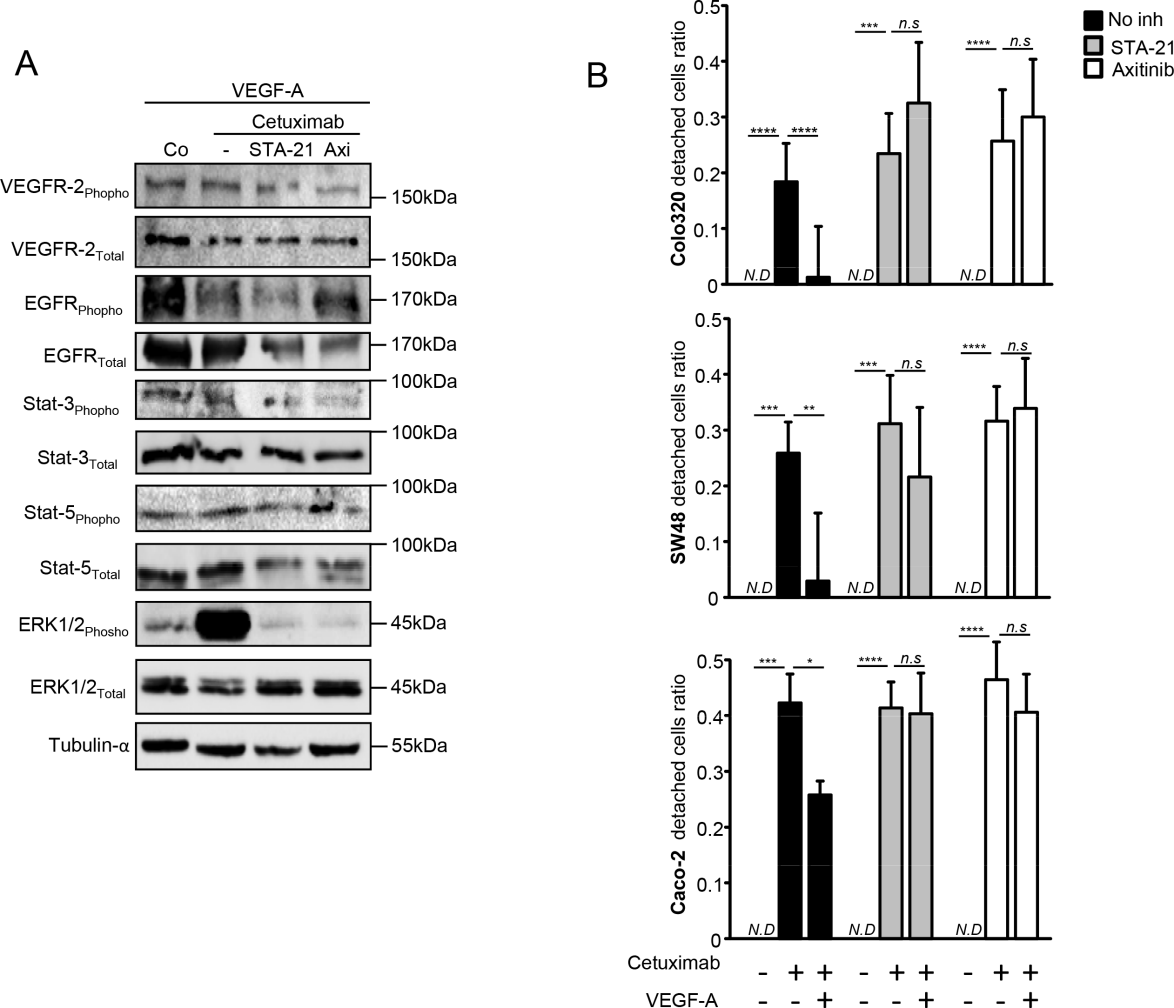
VEGFR2/Stat-3 pathway is involved in VEGF-A-induced resistance to anti-EGFR therapy (**A**) Western blot analysis performed on SW48 colon cancer cell line. Cells were stimulated with human recombinant VEGF-A (5 ng/mL) and with or without axitinib or STA-21 during 24 hours. Cetuximab was added the following day for 24 hours. α-tubulin was used as loading control (Co: Control). (**B**) Cell proliferation analyzed by crystal violet staining. Colon cancer cell lines were incubated or not with human recombinant VEGF-A (5 ng/mL) and STA-21 (10 μM) or axitinib (500 pM) were concomitantly added. Cetuximab (500μg/mL) was added the following day in culture medium and cell death was analyzed 7 days later. (*p < 0,1; **p < 0,01; ***p < 0,001; ****p < 0,0001; *N.D*: not determined, *n.s*: not significant, ANOVA test).

While pretreatment with bevacizumab limits the clinical efficacy of anti-EGFR therapy, we raise the hypothesis that bevacizumab could modify tumor biology and confer resistance to anti-EGFR therapy. Gordon et al. reported that an intravenous injection of bevacizumab led to an increase in serum total VEGF-A in clinical trials, while free VEGF-A concentration was reduced [16]. Since then, other groups have reported counterintuitive increases in the plasma VEGF-A level following bevacizumab administration [17–19]. We also found an increase in VEGF-A serum level in 25 patients obtained from an independent cohort, treated with bevacizumab combined to bi-chemotherapy (FOLinic acid Fluorouracil OXaliplatin or FOLFOX) as a first-line for metastatic colorectal cancer fifteen days after bevacizumab injection. No significant change in VEGF-A serum level was observed in 12 patients suffering from digestive cancer and not treated with bevacizumab as control (Figure 3). To assay the effect of VEGF-A on anti-EGFR therapy *in vitro*, we selected three colorectal cancer cell lines (Colo320, SW48, and Caco2) with a *KRAS*, *NRAS*, *HRAS* and *BRAF* wild-type status. We first checked VEGFRs and EGFR expression by western blotting in those different cell lines (Figure 4A). Cetuximab was able to decrease cell proliferation and cell death *in vitro* (Figure 4B and 4C) by inhibiting EGFR phosphorylation (Supplementary Figure 1). Treatment of tumor cells with recombinant VEGF-A prior anti-EGFR therapy conferred resistance to cetuximab (Figure 4B and 4C). Previous reports showed that VEGF-A exposure induces VEGFR-2 phosphorylation and subsequent Stat-3 activation leading to resistance to apoptosis [20, 21]. Western blot analysis of SW48 and Colo320 cell line showed that VEGFR-2 and Stat-3 are phosphorylated upon VEGF-A treatment (Supplementary Figures 2 and 3). As a result, survival signals such as phosphorylated Erk1/2 are upregulated in SW48 model. Only association of cetuximab and inhibitors such as axitinib for VEGFR-2 and STA-21 for Stat-3 abrogated this pathway (Figure 5A). Although a weak and non-significant axitinib toxicity was observed on Colo320 and SW48 cell lines (Supplementary Figure 4), we clearly demonstrated that VEGFR-2 or Stat-3 inhibition using respectively axitinib or STA-21 resulted in restoration of SW48, Caco-2 and Colo320 sensitivity toward anti-EGFR therapy, when cells were stimulated with VEGF-A (Figure 5B).

All in all, these data underline that VEGF-A exposure confers resistance to cetuximab therapy via VEGFR-2 and Stat-3 activation.

## DISCUSSION

Patients with metastatic colorectal cancer treated with anti-EGFR with or without chemotherapy usually develop resistance within 6 to 12 months after the beginning of the therapy. However, very few studies evaluate the importance of a previous treatment on the efficacy of anti-EGFR therapy. In our retrospective database, we observed that a previous anti-VEGF therapy decreases anti-EGFR efficacy in metastatic colorectal cancer with a wild type status for *KRAS* and *NRAS* genes. Patients treated with bevacizumab before anti-EGFR therapy had a poorer PFS on anti-EGFR therapy compared to patients without a previous anti-VEGF therapy. However, no difference was observed on OS.

VEGFR and EGFR are membrane receptors involved in two independent signaling pathways with specific ligands and specific downstream pathways but are however closely interconnected. Recent reports [22] underline that activation of EGFR pathway can promote neoangiogenesis by up-regulating VEGF-A production or other key proangiogenic mediators. EGF and TGF-α, two ligands of EGFR can induce VEGF-A expression via activation of EGFR in cell culture models and as a consequence could have proangiogenic properties [23]. In preclinical models, EGFR blockade using monoclonal antibody cetuximab, resulted in a down-regulation of proangiogenic mediators, including VEGF-A, interleukine-8 (IL-8), and basic Fibrinogen Growth Factor (FGF) [24]. Such reduction of proangiogenic factors is associated with a reduction in the number of microvessels and metastases. Data from *in vitro* and *in vivo* studies reviewed by Ellis *et al*., suggest that at least a part of the antitumor effect of cetuximab is mediated by inhibition of angiogenesis via a downregulation of proangiogenic molecules [25]. Similar results have also been reported for small-molecule tyrosine kinase inhibitors targeting EGFR, such as gefitinib [25]. However, EGFR inhibition does not completely block VEGF-A production, suggesting that anti-EGFR and anti-VEGF therapies may be synergistic.

On the other hand, angiogenesis may contribute to resistance to anti-EGFR. Preclinical data suggest that an increase in VEGF-A expression and other angiogenic factors can play a role in resistance of anti EGFR-therapy. Viloria-Petit *et al.* showed that variants of A431 lung cancer cells resistant to anti-EGFR, present a higher expression of VEGF-A [26]. This group also showed a direct correlation between the level of VEGF-A and resistance towards anti-EGFR therapy. This is in accordance with our *in vitro* results. Here we show that VEGF-A directly confers resistance to cetuximab via Stat-3 and VEGFR2 activation. Blocking these pathways using VEGFR2 inhibitor axitinib or Stat-3 blocker STA-21, restore tumor cell sensitivity to cetuximab therapy. Such data raise the hypothesis that high level of VEGF-A restrains sensitivity to anti-EGFR and clearly demonstrates the logic to combine VEGFR and EGFR inhibition to provide complementary anti-tumor effects.

A pharmacokinetic model suggests that the intravenous injection of bevacizumab leads to an increase in VEGF-A serum concentration in patients suffering from cancer [27]. This higher VEGF-A serum level is a consequence of inter-compartmental exchange of VEGF-A, due to the formation with the anti-VEGF agent of a VEGF/anti-VEGF complex. These results suggest that a fraction of the anti-VEGF drug moves from blood vessel, allowing the agent to bind the interstitial VEGF-A. When the complex of VEGF/anti-VEGF moves to blood and lymphatic vessels and dissociates, VEGF-A is released and its concentration increases in the plasma. This model shows that rather than depleting VEGF-A in bloodstream, bevacizumab may act by depleting VEGF-A from the tumor interstitium, to release it in the blood [27]. We also confirm in a small cohort of 26 metastatic colorectal cancer patients treated as first-line with bevacizumab combined to chemotherapy, that VEGF-A serum level rapidly increased after bevacizumab injection. Such model raises the hypothesis that bevacizumab therapy could favor anti-EGFR resistance via an increase in the level of serum VEGF-A.

The dual blockade was previously tested in clinical trials. In metastatic colorectal cancer a randomized phase II trial BOND2 examined the efficacy and safety of concurrent administration of bevacizumab plus cetuximab with and without irinotecan, in irinotecan-refractory disease. In this trial, in a population of patients without RAS selection, Saltz *et al.* showed that the addition of bevacizumab to the cetuximab treatment produced a 37% response rate with a median time to progression of 7,9 months [28]. These results are more relevant compared to the results of previously tested association of cetuximab plus irinotecan which give 22% of response rate and 4.5 months of progression free survival [10]. However, two recent phase III trials have shown no benefit for the double biologic combination targeting EGFR and VEGF when used with chemotherapy as first-line therapy for mCRC. In the CAIRO-2 (Capecitabine, Irinotecan, and Oxaliplatin in Advanced Colorectal Cancer) study, 755 front-line mCRC patients were randomized to receive capecitabine/oxaliplatin plus bevacizumab with or without cetuximab. The addition of cetuximab was associated with a poorer PFS (median, 9.4 months vs. 10.7 months; *P* = 0.01) and higher rates of grade 3–4 toxicity (82% vs. 73%; *P* = .006) [29]. Likewise, in the PACCE trial (Panitumumab Advanced Colorectal Cancer Evaluation), 1053 front-line mCRC patients treated with either oxaliplatin-based or irinotecan-based chemotherapy were randomized to receive either bevacizumab alone or combined with panitumumab. The panitumumab arm was also associated with a poorer PFS (median, 10.0 months vs. 11.4 months; *P* < .05) and more grade 3–4 toxicity (90% vs. 77% in the oxaliplatin stratum) [30]. A retrospective evaluation of the CAIRO-2 trial indicated that patients with tumors bearing mutated *KRAS* who received cetuximab, exhibited a poorer PFS compared to the non-cetuximab arm [29]. For the PACCE trial, a retrospective evaluation demonstrated adverse outcomes for the panitumumab arm in tumors with both wild-type and mutant *KRAS* [30]. The results of these phase III trials suggest that the addition of anti-EGFR antibodies is not likely to enhance the effectiveness of bevacizumab plus chemotherapy when used as first-line therapy for patients with mCRC irrespective of *KRAS* mutation status.

This can be explained by the observation that bevacizumab may affect cetuximab distribution in a preclinical model and may limit its efficacy as a consequence [31]. Another explanation comes from a pharmacokinetic model which suggests that the intravenous injection of bevacizumab leads to an increase in VEGF-A serum concentration in patients suffering from cancer [27]. This increase in VEGF-A serum level is a consequence of inter-compartmental exchange of VEGF-A, due to the formation with the anti-VEGF agent of a VEGF/anti-VEGF complex. These results suggest that a fraction of the anti-VEGF drug moves from blood vessel, allowing the agent to bind the interstitial VEGF-A. When the complex of VEGF/anti-VEGF moves to blood and lymphatic vessels and dissociates, VEGF-A is released and its concentration increases in the plasma. This model shows that rather than depleting VEGF-A in the blood, bevacizumab may act by depleting VEGF-A from the tumor interstitium, to release it in the blood [27]. We also confirm in a small cohort of metastatic colorectal cancer patients treated as first-line with bevacizumab combined to chemotherapy, that VEGF-A serum level rapidly increased after bevacizumab injection. Such model raises the hypothesis that bevacizumab therapy could favor anti-EGFR resistance via an increase in the level of serum VEGF-A.

To conclude, our clinical data stress that bevacizumab administration as a first-line may negatively impact on further anti-EGFR efficacy. These results give a biological rational to clinically use anti-EGFR as a first-line of RAS wild-type metastatic colorectal cancer treatment. An alternative bi-chemotherapy without additional bevacizumab treatment may also be planned as first-line. However, benefits observed in PFS are not translated in OS in our study. These results have to be interpreted carefully by taking into account the low number of patients and the retrospective design of this study.

Our *in vitro* models biologically support our clinical data and propose that an increase in VEGF-A serum level after a previous treatment by bevacizumab may be responsible for resistance to anti-EGFR therapy. This may explain previous clinical data showing failure of cetuximab and bevacizumab combination. These results underline the hypothesis that anti-EGFR and anti-VEGF combination therapy could be evaluated using new anti-VEGFR strategy such as axitinib. One can speculate that the use of downstream blockers of VEGFR could be relevant whatever the circulating VEGF-A level.

Another possibility of combined therapy would be to test whether VEGF-A trapping using aflibercept may be efficient in association with anti-EGFR. Aflibercept should limit VEGF-A bloodstream redistribution compared to bevacizumab as the dissociation constant for VEGF-A of aflibercept is up to 500 times lower compared to bevacizumab [32]. We suggest that the complex aflibercept/VEGF-A could be stronger and that VEGF-A serum level will not be increased. These speculations have to be evaluated in clinical trial to be validated.

## MATERIALS AND METHODS

### Patients and methods

We used our database of 399 patients treated for a metastatic colorectal cancer at Georges Francois Leclerc Cancer Center from January 2001 to December 2013. In this database we selected all patients (198) treated as second or third-line by cetuximab or panitumumab alone or in association with chemotherapy. We completed molecular biology testing if not performed previously and tested tissue samples for *KRAS* (exons 2, 3 and 4), *NRAS* (exons 2, 3 and 4). We only included 128 patients treated with anti-EGFR therapy with a *KRAS* and *NRAS* wild type status. The following data were collected and analyzed: age, performance status (according to WHO criteria) at the time of the first cycle, gender, primary tumor site (colon or rectum), localization of metastatic sites, previous anticancer drugs received, CEA and Lactate Deshydrogenase (LDH), Alkaline Phosphatase level and leukocytes count at the time of the first cycle, the type of chemotherapy used with anti-EGFR chemotherapies. Progression-free survival on anti-EGFR therapies and overall survival were also recorded. This study was approved by the local scientific and ethics committee.

### *KRAS*, *NRAS* mutation analysis

DNA was extracted from paraffin-embedded colorectal cancer samples after histological control (Hematoxylin-Eosin-Saffron) for at least 50% tumor cells. *KRAS* exons 2 (codons 12 and 13), 3 (codons 59 and 61) and 4 (codons 117 and 146) and *NRAS* exons 2 (codons 12 and 13), 3 (codons 59 and 61), and 4 (codons 117 and 146) were investigated using direct sequencing by Sanger methods or allelic discrimination.

### Clinical data statistical analysis

All patients were followed up until death or the end of data recording (December 31st 2013). Progression free survival on anti-EGFR therapy was calculated from the date when the therapy started to the date of disease progression, and overall survival was calculated from the date of the beginning of treatment for the metastatic disease to the date of death. Median follow-up with its 95% confidence interval (CI) was calculated using the reverse Kaplan-Meier method. Patient or disease characteristics were examined using the Chi2 test or Fisher's exact test for qualitative variables, and the Student t or Mann-Whitney tests for continuous variables, as appropriate to compare the group of patient treated with bevacizumab in first line or not. Survival probabilities were estimated using the Kaplan-Meier method and survival curves were compared using the log-rank test. A multivariate Cox model was used to estimate the effects of a previous bevacizumab regimen on progression free survival and on overall survival after adjusting with clinical parameters selected in univariate analysis. In the multivariate model only variable with *p* < 0.1 in the univariate model were retained. The level of significance to retain a variable was set at *p* < 0.05.

Statistical analyses were performed using MedCalc Software. All tests were two sided, and *P* values < 0.05 were considered statistically significant.

### *In vitro* procedures

### VEGF-A assay

Twenty six patients from another cohort (Bevacapi study NCT01810777) were selected. Patients were all suffering from mCRC and treated with bichemotherapy FOLFOX combined to bevacizumab as first-line. Another 12 patients suffering from digestive cancer and not treated with anti-VEGF therapy were also selected. For both groups, blood was sampled before and 15 days after first chemotherapy administration. After sera preparation, VEGF-A was quantified and analyzed by ELISA (eBiosciences, Rennes, France) using manufacturer's protocol. Samples were analyzed in triplicate.

### Cell culture

SW48, Caco-2 and Colo320 human colon cancer cell lines were obtained from the American Type Culture Collection (ATCC). Cells were grown in DMEM 4, 5 g/L glucose (Lonza, Levallois, France) supplemented with 10% (vol/vol) fetal bovine serum (FBS; Lonza) in an atmosphere of 95% air and 5% CO_2_ at 37°C.

### Crystal violet staining

Colon cancer cell lines were obtained from American Tissue Culture & Collection and selected for *in vitro* assay due to their *KRAS*, *NRAS* wild type status. Cells (2,000 cells/well) were seeded in a 96-well plate in appropriate medium. Twenty-four hours later, cells were treated with 500 μg/mL cetuximab (Merck, Darmstadt, Germany), and incubated for another 7 days. After treatment, cells were washed two times with Phosphate Buffered Saline (PBS) and fixed with 100% ethanol for 30 minutes before crystal violet staining. Crystal violet was then suspended in 33% acetic acid and OD was read at 590 nm with a Wallac 2 spectophotometer (Perkin-Elmer, Villebon sur Yvette, France). In some cases cells were treated with human recombinant VEGF-A (Peprotech, Neuilly sur Seine, France) at 0,5 or 5 ng/mL the day before cetuximab treatment. In other cases STA-21 10 μM (Bertin Pharma, Montigny le Bretonneux, France) and axitinib 500 pM (Abcam, Cambridge, UK) were used concomitantly with VEGF-A stimulation. The following day, cetuximab was added for seven days in media containing or not inhibitors. Data displayed are means and SD from 3 independent experiments. Ratio of detached cells were calculated using the following formula *R* = 1-(ODx/ODco). Statistical analyses were performed using GraphPad Prism software.

### AnnexinV/7AAD staining

Cells were treated with or without VEGF-A and inhibitors. The following day, cetuximab was added in the media containing inhibitors. 24 hours after, cells were harvested and stained using AnnexinV FITC and 7AAD kit (BDPharmingen, Le pont de Claix, France) with manufacturer's protocol. Signal was measured with LSRII Cytometer (BDPharmingen) and analyzed with FlowJo software (Flowjo LLC, Ashland, OR, USA). Data displayed are means and SD from 3 independent experiments. Statistical analyses were performed using GraphPad Prism software.

### Western blot analysis

Cells were seeded in a 6 well plate in appropriate medium containing 10% FBS. After cells reached 80% confluence, recombinant VEGF-A wad added to medium. Whole-cell lysates were prepared as described previously, [32] by lysing the cells in boiling buffer (1% SDS, 1 mM sodium vanadate, 10 mM Tris (pH 7.4)) in the presence of complete protease inhibitor mixture. The viscosity of the samples was reduced by sonication. Whole-cell lysate samples were separated by SDS-polyacrylamide gel electrophoresis, and electroblotted to a nitrocellulose membrane (GE Healthcare, Villacoublay, France). After incubation for 1 h at RT by 5% nonfat milk in phosphate-buffered saline–0.1% Tween-20, membranes were incubated overnight with indicated primary antibody diluted in Tris-buffered saline-BSA5%–Tween-20, washed, incubated with the secondary antibody for 30 min at RT, and washed again before analysis with a chemiluminescence detection kit (Amersham, Villacoublay, France) and chemidoc acquisition system (Biorad, Marne la Coquette, France). The following Abs were used: Stat-3 (#4904), p-Stat-3 (Y705) (#9145), VEGFR1 (#2893), VEGFR2 (#2479), p-VEGFR2 (#4991), Stat-5 (#9363), p-Stat-5 (#4322), Erk1/2 (#9102) and p-Erk1/2 (#9101) (all from Ozyme, Saint Quentin en Yvelines, France); EGFR (ab52894), p-EGFR (ab40815) and α-tubulin (ab15246) (all from Abcam, Paris, France); p-VEGFR2 (#07–722) (from Merck Millipore, Fontenay sous-Bois, France); HSC-70 (sc7298) (from Santa Cruz, Heidelberg, Germany). Secondary Abs HRP-conjugated polyclonal goat anti-mouse and swine anti-rabbit immunoglobulins (Jackson ImmunoResearch, Interchim) were also used. Images displayed are representative data out from three independent experiments. Quantifications were obtained using quantity tools thumbs provided Image Lab Software (Biorad, Marne la Coquette, France).

## SUPPLEMENTARY MATERIALS FIGURES


