# Supplementary material for: Does bevacizumab impact anti-EGFR therapy efficacy in metastatic colorectal cancer?
Source: Oncotarget. 2016 Jan 25;7(8):9309–21. doi: 10.18632/oncotarget.7008 (PMC4891042; doi:10.18632/oncotarget.7008)
Supplement: Supplementary file 1 [file oncotarget-07-9309-s001.pdf]

Does bevacizumab impact anti-EGFR therapy efficacy in metastatic colorectal cancer?

Supplementary Materials

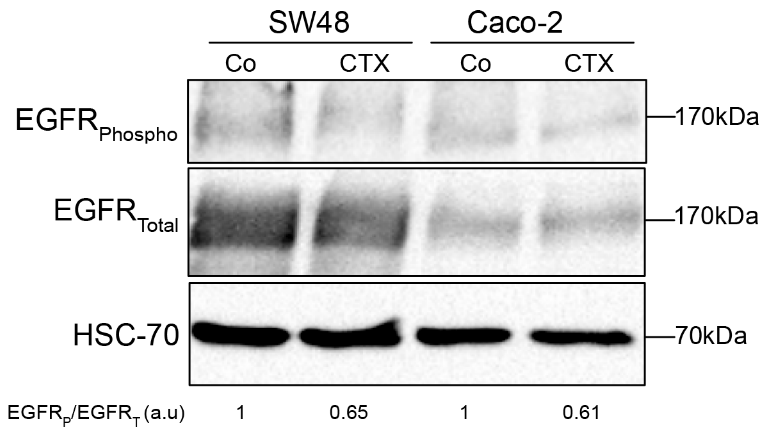

Supplementary Figure S1: SW48 and Caco-2 cells were treated 24 hours with cetuximab (CTX) at 500 µg/mL. Western Blotting analyses show a decrease in EGFR activation. HSC-70 was used as loading control. Numbers displayed beneath images represent band quantification in arbitrary unit (a.u).

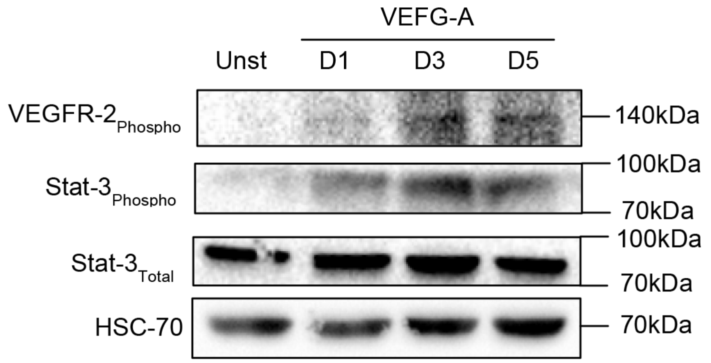

Supplementary Figure S2: Western blot analysis performed on Colo320 colon cancer cell line. Cells were stimulated with human recombinant VEGF-A (5 ng/mL) during indicated times. HSC-70 was used as loading control. (Unst: unstimulated, D: Day).

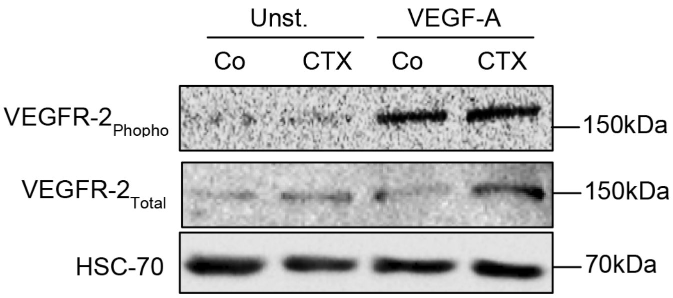

Supplementary Figure S3: SW48 were stimulated or not with 5 ng/mL with VEFG-A during 24 hours. Cells were then treated or not 24 hours with cetuximab at 500 µg/mL. Western Blotting analyses show an increase in VEGFR-2 activation with VEGF-A. HSC-70 was used as loading control. (Unst: Unstimulated; Co: Control; CTX: Cetuximab).

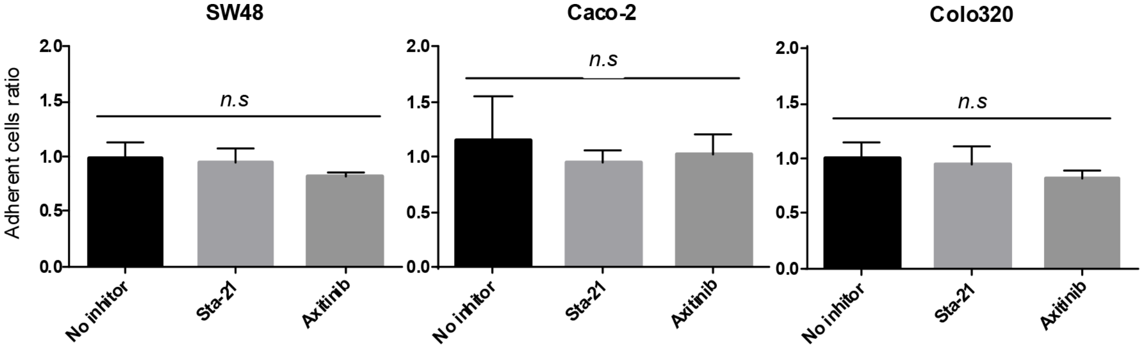

Supplementary Figure S4: Adherent cells were quantified using crystal violet staining. No significant toxicity was observed with Stat-3 blocker (Sta-21) or with VEGFR-2 inhibitor (Axitinib). (n.s: not significant, ANOVA test).
